# Developing Technology for the Production of Innovative Coatings with Antioxidant Properties for Packaging Fish Products

**DOI:** 10.3390/foods12010026

**Published:** 2022-12-21

**Authors:** Hana Derbew Gedif, Joanna Tkaczewska, Ewelina Jamróz, Marzena Zając, Mirosław Kasprzak, Paulina Pająk, Wiktoria Grzebieniarz, Nikola Nowak

**Affiliations:** 1 Department of Animal Product Technology, Faculty of Food Technology, University of Agriculture, ul. Balicka 122, 30-149 Kraków, Poland; 2 Department of Food Engineering, Faculty of Chemical and Food Engineering, Bahir Dar Institute of Technology, Bahir Dar 26, Ethiopia; 3 Department of Chemistry, University of Agriculture, ul. Balicka 122, 30-149 Kraków, Poland; 4 Department of Food Analysis and Quality Assessment, Faculty of Food Technology, University of Agriculture in Kraków, ul. Balicka 122, 30-149 Kraków, Poland

**Keywords:** shelf-life, furcellaran, active coatings, fish by-product

## Abstract

In this study, we investigated the effects of furcellaran–gelatine (FUR/GEL) coatings incorporated with herb extracts on the quality retention of carp fish during refrigeration. Nutmeg, rosemary, thyme, milfoil, marjoram, parsley, turmeric, basil and ginger were subjected to water and ethanol extraction methods (10% concentration of herbs). The water extractions of the rosemary and thyme (5%) were used for the further development of coatings due to their high 2,2-Diphenyl-1-picrylhydrazyl (DPPH: 85.49 and 83.28%) and Ferric Reducing Antioxidant Power Assay values (FRAP: 0.46 and 0.56 mM/L) (*p* < 0.05), respectively. A new, ready-to-cook product with the coatings (carp fillets) was evaluated regarding quality in terms of colour parameters, texture profile, water activity, Thiobarbituric Acid Reactive Substances (TBARSs) and sensory analyses during 12 days of storage at 4 °C. The results show that the colour of the carp fillets treated with the rosemary and thyme extracts became slightly darker and had a propensity towards redness and yellowness. In contrast to the control group, the carp fillets stored in the coatings with the rosemary extract effectively slowed the lipid oxidation processes. Therefore, the innovative coatings produced from carp processing waste may have high potential as components in convenience food products and could extend the shelf-life of carp fillets during refrigerated storage. However, further research is needed to assess the microbiological stability of the obtained food products.

## 1. Introduction

The use of bio-based coatings as well as the preservation of films are currently viewed as effective and eco-friendly methods in the maintenance of food product freshness while contributing to shelf-life extension [1]. Food products have been successfully preserved using edible coatings that contain a variety of bioactive compounds [2]. The main component of biopolymer coatings can be derived from food processing by-products or underused proteins, lipids or polysaccharide sources that are biodegradable and edible [3]. Bioactive compounds are usually isolated from natural sources such as algae, lichens and higher plants, as well as mushrooms. These compounds have attracted a great deal of attention in the scientific field. Plant extracts demonstrate a broad spectrum of active properties and relatively low toxicity while not causing significant side-effects on human health [4,5]. Waste from fish processing offers great potential in the recycling of by-product materials and their conversion into relevant products with a nutritional or functional value. Fish gelatine products have broad application in the preparation of edible coatings and films; however, those derived from pure gelatine demonstrate weak biological features, i.e., antioxidant- and antimicrobial-related abilities [6]. To date, various polysaccharides have been used as components of gelatine–polysaccharide films and coatings [7,8]. Nonetheless, the use of furcellaran as a component of such coatings has not yet been extensively investigated and is still quite novel. Furcellaran is a negatively charged polysaccharide obtained from the red algae *Furcellaria lumbricalis*. It is a linear polymer made up of the following components: (1 → 4)-3.6-anhydro- α-D-galactopyranose and a fragment of (1 → 3) β-D-galactopyranose with a sulphate group at C-4. Furcellaran, along with carrageenan, is classified as a food additive with the E 407 code that is accepted by the European Commission (2011) [9]. Fish gelatine and furcellaran are compatible due to the formation of electrostatic interactions while the two macromolecules are oppositely charged in appropriate pH environments [9].

Lipid oxidation is one of the primary causes of food deterioration. It has negative impact on the sensory qualities of products, causing rancidity, the generation of harmful aldehydes and depletion of nutritional content [10,11]. Food products with high lipid content, such as fish oils, other fish products, and meat products, are particularly vulnerable [10]. Numerous synthetic antioxidants have been used to delay lipid oxidation; however, their use is highly regulated because of the possible health risks they pose. The carcinogenic effects of some of these compounds have been revealed, and their use is declining. Thus, there is a demand for effective natural preservatives to replace synthetic compounds [12]. The addition of natural antioxidants derived from herbal extracts as a way of increasing the shelf-life of food products has become increasingly popular. It has also improved the stability of lipids and lipid-containing foods, thereby preventing sensorial and nutritional quality loss [13]. Many herbal extracts have antioxidant activity due to their polyphenolic, phenolic acid, biological and anticoagulant compounds. These constituents have made it possible to replace chemical preservatives with them. They are also adequate for daily consumption. The method applied for their extraction and solvents affect their properties [14].

Future research should be focused on creating new technologies to enhance the carrier properties of edible coatings and assess the effectiveness of bioactive compounds. Nowadays, the application of such edible films and coatings is limited. One of the most significant barriers is cost, which limits their use to high-value products. The absence of materials with the required functionalities, the cost of investment for the installation of new coating equipment and the difficulty of the manufacturing process are additional limitations in the commercial use of edible films and coatings. Furthermore, more research is needed to develop new edible films and coatings incorporating active components to recognise interactions between the ingredients used in their production [3].

It is noteworthy that in this work, we were the first to use gelatine obtained from carp skins as a solution to extract dry herbs from thyme or rosemary. The procedure does not require the preparation of additional herbal extracts or the drying of the gelatine solution. It can be used with dry herbs and by implementing them into a gelatine solution. There are many works in which a previously prepared herbal extract, e.g., water or alcohol, had been added to the created biopolymer coating [15,16,17]. However, it is noteworthy that in the case of this solution, we additionally reduced the time and costs of preparing such an extract.

To date, various polysaccharides have been used as components of gelatine–polysaccharide films and coatings [2,7,8]. Nevertheless, this is the first study in which coatings containing a furcellaran–gelatine complex were made directly from carp skins in the form of a solution. Therefore, the primary objective of our study was to develop technology for the production of coatings made of protein waste from carp processing that can be used as components in healthy convenience food products. The secondary objective of the study was to select the types of herbs that could be used as active ingredients and to identify the herb extraction method that obtains a coating with the highest possible antioxidant activity. The innovative coatings were applied to fish fillets, and their quality was assessed throughout the storage period. The designed method is expected to be simple and more feasible for use at small fish processing units on fish farms.

## 2. Materials and Methods

### 2.1. Selection of Herbs as Active Ingredients for Coatings and Choice of Extraction Method

#### 2.1.1. Plant Material

Dry plant materials from the herbarium include basil (*Ocimum basilicum*) and nutmeg (*Myristica fragrans Houtt*.) stones and leaves; milfoil (*Millefoliflos*) and rosemary (*Rosmarinus officinalis*) flowers; the aerial part of parsley (*Petroselinum crispum*); marjoram (*Maiorana hortensis*); thyme (*Thymus vulgaris*); turmeric (*Curcumae longae*); and ginger (*Zingiber boehm*), rhizoma. All dry plants were purchased from an ecological market (Kraków, Poland).

#### 2.1.2. Preparation of Ethanol and Water Extracts of Herbs for Assessing Antioxidant Activity

The 20 g sample of dried herbs comprised a mixture of different individual plants and their various parts. Herbal samples were taken randomly. A 10 g herb mixture was added to 100 mL ethanol (analytical grade) and sonificated while being stirred in a dark glass vessel for 2 h at a temperature of 40 °C. This was marked as the ethanol herb extract. The resulting ethanol extracts were obtained after filtering the mixture on filter paper and then refrigerated until further analysis.

The next 10 g of the herbs were subjected to water extraction using the same concentration of herbs in water (100 mL) and were kept in a magnetic stirrer for 2 h at a temperature of 90 °C. This was designated as the water plant extract. The resulting plant extracts were obtained after filtering the mixture on filter paper, which was then refrigerated until further analysis.

#### 2.1.3. Antioxidant Analysis of Herbal Extracts

##### Ferric Reducing Antioxidant Power (FRAP)

The reducing power of the 1% extract was measured using the method described by Jamróz et al. [18] with some modifications. The herbal extract (0.4 mL) was then incubated with the FRAP reagent (3.6 mL) comprising 10 mM TPTZ (2,4,6-tripyridyl-s-triazine) and 20 mM FeCl3 at 37 °C. A Helios Gamma UV-1601 spectrophotometer (Thermo Fisher Scientific, Waltham, MA, USA) was used to measure absorbance at 595 nm after 30 min. The results are expressed as mM/L Fe_2_SO_4_.

##### DPPH Radical Scavenging

The samples (0.1% extract) were mixed with 0.1 mM *DPPH*• in an ethanol solution at a 0.1:2.9 ratio (*v/v*) for the *DPPH*• assay. The cells were incubated in the dark for 30 min. The solution’s absorbance (*A_sample_*) was evaluated at 517 nm (Helios Gamma, Thermo Fischer Scientific, Waltham, MA, USA) and compared to the blank, in which the film extract was replaced with distilled water (*A_blank_*). *DPPH radical scavenging* activity was determined using the following equation:

(1) $$DPPH\;radical\;scavenging\;\left(\%\right)=\frac{A_{blank}-A_{sample}}{A_{blank}}\times 100\%$$

### 2.2. Preparation of Coatings

Gelatine solution: Carp skins (*C. carpio*) were obtained from a local fish processing plant (Sona Sp. z o. o. Koziegłówki, Poland). Gelatine was obtained via an extraction procedure. Briefly, 300 g of carp skin was minced and soaked at room temperature in 1500 mL of the following water solutions: 0.05 M sodium hydroxide, 0.004 M hydrochloric acid and 0.05 M citric acid. The carp skin was immersed for 90 min in each of the solution treatments and intermediately washed with tap water. The skin was then rinsed again with water, mixed with 1500 mL of Milli-Q water and incubated overnight at 45 °C. The resulting material was then passed through a sieve (72 µm diameter) to remove any leftover skin from the gelatine solution. The gelatine solution was used to prepare a coating without drying.

Extraction of rosemary and thyme polyphenols: Based on the obtained antioxidant properties of 9 herbs tested in water or ethanol solutions, the water extractions of thyme and rosemary were selected. Thus, we decided that the herb could be extracted directly into the gelatine solution, without additional water extraction. Thyme (*Thymus pulegioides*) and rosemary (*Rosmarinus officinalis*) were cultured in Poland during the summer of 2021. In September 2021, they were picked, dried and vacuum-packed. The gelatine solution with a 5% addition of dry thyme or rosemary (g/mL) was prepped and then incubated at a temperature of 85 °C for a 2-hour period. Following incubation, the solution was subjected to filtration, which was achieved using a sieve (with a 72 µm diameter). The solution’s pH was adjusted to 7. After adjustment, the solution was applied as a component for coating.

Fabrication of coatings: Type 7000 furcellaran with M_w_ 2.951 × 10^5^ was chosen and procured from Est-Agar AS (Karla Village, Estonia). The furcellaran contained 79.61% carbohydrates, 1.18% proteins and 0.24% fat. The solution containing herb–gelatine/furcellaran was prepared in the following manner: 3.5% of the furcellaran (g/mL) sample was mixed with the gelatine and herb addition at a ratio of 3:7. This was carried out in defined conditions (i.e., 400 rpm mixing rate, temp. at 50 °C, for 30 min). As a plasticiser, glycerine was utilised in a 1 mL volume, and it was further added to a 100 mL sample of the solution consisting of furcellaran and gelatine.

**Figure 1:**
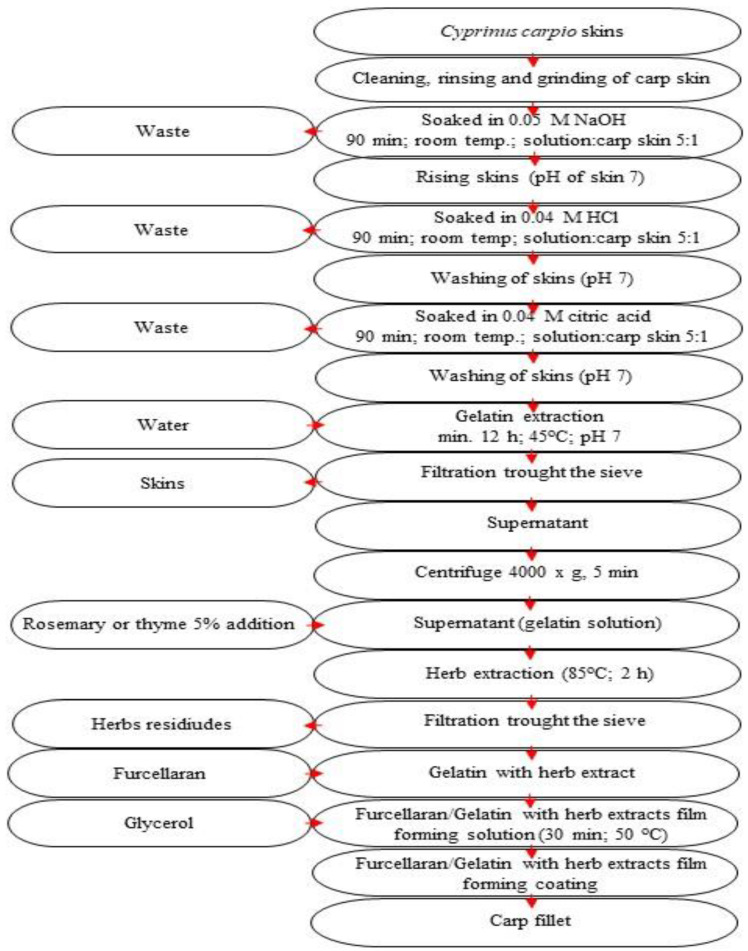
Scheme of innovative coating preparation.

After several technological operations, as presented in Figure 1, a final coating material was obtained.

Due to the high antioxidant activity of rosemary—or thyme-rich water extracts—gelatine in water solutions (produced by conversion of collagen with heat treatment) was further used as a solution to create extracts from dry thyme or rosemary for coating fabrication. Without the preparation of additional herbal extracts, the procedure continued with a mixture of a gelatine water solution with herbs, followed by subsequent steps, as previously described.

### 2.3. Properties of Antioxidant Coatings

#### 2.3.1. Total Phenol Content (TPC) in Coatings

The TPC in the coatings was quantified using the Folin–Ciocalteu colourimetric method according to Oriani et al. [19]. A 10% coating solution (125 μL) and 0.5 mL of water were mixed with 125 μL of the Folin–Ciocalteu reagent; after 6 min, 1.25 mL of sodium carbonate solution (7%) was added. Water was then added to a final volume of 3 mL. After 120 min in the dark and at room temperature, the absorbance was measured at 760 nm, while the total phenols were quantified using a gallic acid calibration curve.

#### 2.3.2. Antioxidant Activity of Coatings

The coatings were heated to 45 °C before being diluted to 10% in distilled water. The coated extract tubes were placed in a 50 °C water bath and shaken for 10 min to confirm that the coatings were completely dissolved. The reducing power of the coating extracts was measured using the method described by Khantaphant and Benjakul [20], with some modifications. Then, 0.4 mL of the herbal extract or coating extract was incubated with 3.6 mL of the FRAP reagent comprising 10 mM of TPTZ (2,4,6-tripyridyl-s-triazine) and 20 mM FeCl_3_ at 37 °C. A Helios Gamma UV-1601 spectrophotometer (Thermo Fisher Scientific, Waltham, MA, USA) was used to measure absorbance at 595 nm after 30 min. The results are expressed as mM/L Fe_2_SO_4_.

#### 2.3.3. Ultraviolet–Visible (UV-Vis) Spectroscopy Analysis

UV-Vis analysis was performed using a UV-5500 spectrophotometer (UV 5500 Metash), and the absorbance spectrum was recorded between 200 and 400 nm.

#### 2.3.4. Colour Parameters of Coatings

Colour measurements of the coatings were performed with a Konica Minolta spectrophotometer CM-3500d (Osaka, Japan) using CIELAB colour parameters. The instrument was calibrated on black and white enamel, according to the manufacturer’s guidelines. Analyses were performed in reflectance mode with illuminant D and an observer angle of 10°.

### 2.4. Influence of Tested Coatings on Quality of Refrigerated Carp Fillets

#### 2.4.1. Preparation of Samples

**Figure 2:**
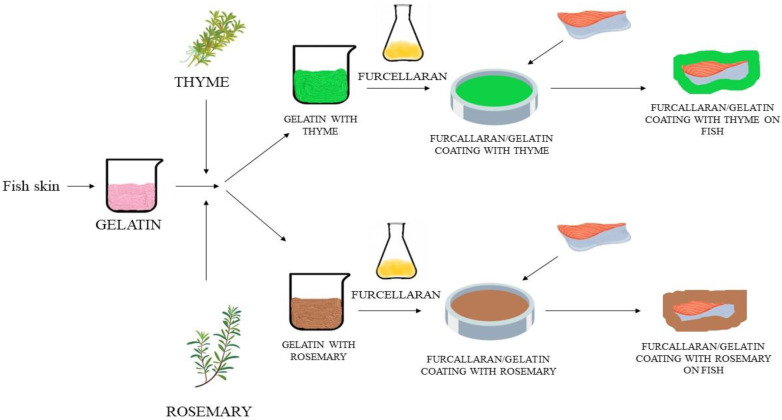
Scheme of the experimental process.

The newly created coatings were tested on raw carp fillets to assess their efficiency. A local fish processing plant (Gospodarstwo Rybackie Przyborów, Przyborów, Poland) provided fresh carp (*C. carpio*). Three carp fillets were randomly selected on each day of the analysis. The fillets were dipped twice in the coating solution and air-dried at room temperature at 10 min. The fish samples were divided into 4 groups: uncovered (K), covered in FUR/GEL coating without herbs (G), covered in FUR/GEL coatings with 5% thyme (T), and covered in FUR/GEL coatings with 5% rosemary (R). The coated fillets were placed on a plastic tray (polyethylene *LDPE*), which was sealed in a heat sealer with synthetic film, and then stored at 4 °C. The experimental scheme is illustrated in Figure 2.

#### 2.4.2. Texture, Colour and Water Activity of the Carp Samples

Colour measurements of the fillets were performed using a Konica Minolta spectrophotometer CM-3500d (Osaka, Japan), applying CIELAB colour parameters. The instrument was calibrated on black and white enamel, in accordance with the manufacturer’s instructions. Analyses were performed in reflectance mode with illuminant D and an observer angle of 10°. Each sample was examined in 3 independent replicates, each with 4 measurements.

A TAX-T2 Plus texturometer was used for instrumental texture analysis (Stable Micro Systems, United Kingdom). The analysis used 7 cm × 2 cm × 2 cm samples penetrated to 50% of the initial height using a P/6 cylindrical probe at a test speed of 2.0 mm/s.

Water activity was assessed using a Novasina LabMaster-aw (Zurich, Switzerland) water activity measuring device based on changes in electrolytic resistance.

#### 2.4.3. Oxidation Rate of Fish Lipids

Thiobarbituric reactive substance (TBARS) analyses were carried out in the manner previously described by Jamróz et al. [21].

#### 2.4.4. Sensory Analysis of Carp Fish Fillets Covered with Coatings

Carp fillets were divided into 100-gram chunks. Each chunk was wrapped in a FUR/GEL coating without herbs (G), FUR/GEL coatings with 5% thyme (T) or FUR/GEL coatings with 5% rosemary (R) and covered with parchment paper. The filets were baked in the oven for 30 min at a temperature of 200 °C, without air circulation. Afterwards, after switching the oven off, the aluminium foil was opened, and the samples were incubated in the oven for another 10 min to remove the excess of water vapor and to dry the surface of the samples. The hot fillets were tested immediately after baking. Sensory evaluation of the carp fillets with herbal coatings was carried out at the Laboratory for Sensory Analysis of the Faculty of Food Technology, University of Agriculture in Kraków, in accordance with the standards of PN-EN ISO 8589:2010. A professional sensory panel of 15 assessors trained according to PN-EN ISO 5492:2009, PN-EN ISO 3972:2016-07, PN-EN ISO 11132:2017-08, PN-EN ISO 10399:2018-03 and PN-EN ISO 8586:2014-03 was employed. The panellists were handed cards with detailed descriptions regarding the typical taste attributes of baked carp fillets. For this purpose, quantitative descriptive analysis (QDA) was used in accordance with the standard Sensory Profiling ISO 13299:2016. Taste attribute intensities (fishy, salty, sweet, sour, bitter, muddy, fish oil, rancid, rosemary and thyme) were presented on a scale from 0 to 5, where 0 means ‘imperceptible’, and 1 means ‘the least intense’ and 5 ‘the most intense’. Before evaluation proper, the panellists were asked to propose scores for the best carp fillet they can imagine. The average scores for those attributes were treated as the ideal carp fillet. Moreover, acceptability evaluation of appearance, smell, texture, taste and overall acceptance was conducted using a 5-point hedonic scale, where 1 means ‘dislike very much‘, 2 means ‘dislike slightly‘, 3 means ‘neither like nor dislike‘, 4 means ‘like slightly‘ and 5 means ‘like very much‘.

The panellists were shown the samples on white plastic plates marked with a 3-digit random code. To minimise the inaccuracy and masking of sensory qualities, distilled water and sugar-free rusks were offered to neutralise the mouth after tasting each item.

### 2.5. Statistical Analysis

Statistical analyses were performed using Statistica v. 13.0 software (Tibco, Palo Alto, CA, USA). Each experiment was performed with 3 independent replicates and 3 repetitions for each replicate. The Shapiro–Wilk test was used to determine normality of the results, whereas variables with non-normal distributions were converted using Box–Cox data conversion. Two-way analysis of variance (ANOVA) was performed to determine the differences between antioxidant properties in various types of herbal extracts and methods of extraction. The selected herb types used for creating the coatings and the results of the antioxidant analysis, depending on the coating, were compared using one-way ANOVA with Tukey’s post hoc test. The results are presented as the mean ± SEM. Data visualisation was performed using the R software package, version 4.2.1 (R core group).

The fish preservation test results were subjected to Shapiro–Wilk tests, and after confirming the normality of the results, two-way ANOVA testing was performed with regard to storage time and coating type for fish coating servings as 2 independent variables. To determine the differences between means, Tukey’s post hoc test was performed with statistical significance set at *p* < 0.05.

## 3. Results and Discussion

### 3.1. Development of Active Coatings

According to the literature, the final step in the conversion of collagen into gelatine involves the breakage of hydrogen bonds by raising the temperature. In a typical industrial practice, the first extraction of mammalian gelatine is made at or above 45 °C followed by extractions at successively higher temperatures [22]. Although the denaturation temperature for fish collagen is much lower, the extraction temperatures for mammalian gelatines have been adopted for the extraction of cold-water fish gelatines [23]. The extraction rate increases with an increase in extraction temperature. The manufacturer must therefore find the optimal ratio between the rapid extraction at high temperature and high gel strength. The extractions at higher temperatures provide gelatine samples with lower gel strength. These effects are caused by hydrolysis, leading to decreasing molecular weights upon an increase in the severity of the extraction conditions [24].

However, there are literature data showing that fish gelatine can be heated at very high temperatures without any negative effect on the obtained results. In the studies conducted by Duan et al. [25], gelatine was obtained from carp skins at 80 °C, and its quality was satisfactory, while its strength was lower compared to that of gelatines obtained at lower temperatures. Furthermore, other researchers also heated fish gelatine to 85 °C to obtain active coatings, which did not deteriorate their quality [26].

Moreover, the obtained coatings are complexes of gelatine and furcellaran. Furcellaran has excellent gel-forming properties and can form strong complexes with gelatines [27].

A coating that was obtained gelled perfectly after it was applied on the product.

#### 3.1.1. Antioxidant Activity of Herbs

**Figure 3:**
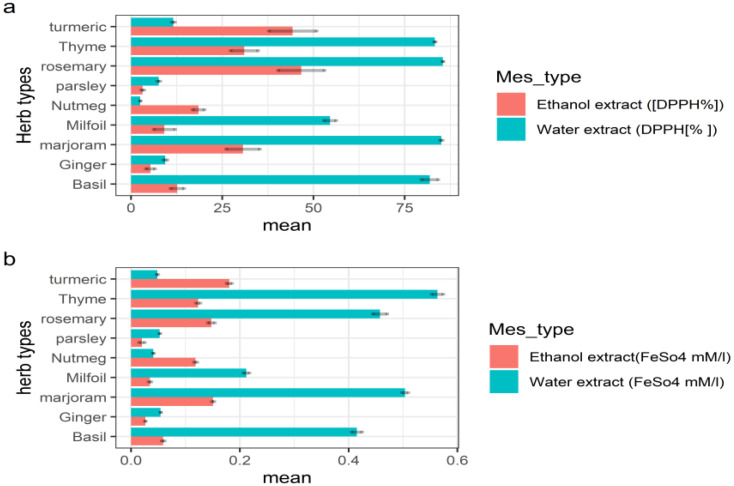
Antioxidant activity of different herb types (mean values ± standard errors). (**a**) is using the DPPH method, (**b**) is using the FRAP method.

The antioxidant activity of an extract is influenced by the extraction process, particularly the applied solvent and temperature [28]. In the current study, ethanol and water were used as solvents for herbal extraction. Evaluation of the antioxidant potential of different herbal extracts provides significant information on their possible use as antioxidant sources. As shown in Figure 3, the water and ethanol extracts of different herb types demonstrated antioxidant properties, as measured using the *DPPH* and FRAP methods.

For most herb types, the water extract was found to have higher antioxidant properties than the ethanol extract. Solvent extraction is the most commonly used method for the isolation of antioxidant components from plants. The presence of various antioxidant compounds with different chemical properties and polarities, which may or may not be dissolved in specific solvents, strongly depends on the nature of the solvent used for extraction [29]. The higher antioxidant activity of plant water than that of ethanol extracts may be due to the fact that ethanol compounds are very often extracted in larger amounts in more polar solvents, e.g., an ethanol/water mixture compared to absolute methanol [30]. There are works in which it is noted that the use of water or ethanol solvent does not significantly affect antioxidant properties of the plant extract [31].Gramza et al. [32] observed that the highest antioxidant potential is found in tea samples extracted with 95% ethanol rather than water, whereas Petlevski et al. [33] compared the type of solvent (water and ethanol) when obtaining an extract from *Pelargonium radula*. They reported that hot water extraction increases the amount of antioxidant metabolites in a given extract without affecting their antioxidant activity. In addition, elevated temperature may contribute to the glycosidic bond hydrolysis of phenolic compounds, increasing the number of phenolic hydroxyl groups and, thus, improving antioxidant activity.

A lack of significant differences between the *DPPH* values was found for rosemary, thyme, basil and marjoram; however, they clearly showed the highest *DPPH* activity. Rosemary exhibited the highest *DPPH* values for the water and ethanol extracts (85.49% and 46.67%, respectively). Similar results were obtained by Ulbin-Figlewicz et al. [34], indicating that a higher *DPPH* free radical scavenging activity (0.129 μM Trolox/mL) was obtained for the rosemary extract in comparison to other herbs. These results are also in accordance with those of another study on the phenolic profile of commonly used culinary herbs and spices conducted by Vallverdú-Queralt et al. [35], who found that rosemary and thyme demonstrated higher *DPPH* antioxidant activity owing to their higher content of phenolic compounds, such as rosmarinic acid, along with other compounds (i.e., caffeic acid and syringic acid). Andrade et al. [36] found that the rosemary ethanolic extract showed the highest antioxidant capacity and the highest amount of carnosol, as well as rosmarinic and carnosic acids, of all analysed herbs. These authors concluded that rosemary has potential as an active ingredient in protein-based films.

The results of FRAP analysis concerning different types of herbs were in the range of 0.56–0.04 mM/L for the water extract and were in the range of 0.18–0.02 mM/L for the ethanol extract. The highest antioxidant activity measured using FRAP analysis of both water and ethanol extracts was detected in thyme and turmeric herbs (0.56 and 0.18 mM/L, respectively). The antioxidant activity of thyme is attributed to antioxidants such as thymol and carvacrol, as well as their dimerisation products, such as biphenyl compounds and flavonoids (quercetin, eriocitrin, luteolin and apigenin) [37].

These results reveal that rosemary and thyme water extracts had the highest antioxidant activity and demonstrated good potential for incorporation into the production of innovative coatings with antioxidant properties for the packaging of fish products. Therefore, thyme and rosemary were chosen for further experimentation and application in coatings.

#### 3.1.2. Antioxidant Activity of Coatings

The antioxidant activities of the obtained coatings are shown in Table 1.

Our results demonstrate that the pure gelatine solution had almost the same very low antioxidant activity as that of coatings without herbs (FRAP value 0.029 mM Fe_2_SO_4_/L). It was observed that the rosemary or thyme polyphenols incorporated into the coatings significantly reduced the ferric ions to ferrous ions (*p* < 0.05) and caused an increase in the antioxidant activity of the obtained coatings. Similar results were reported by Jancikova et al. [38], who found very high antioxidant activity of films developed with the furcellaran–gelatine hydrolysate (GELH) with the rosemary extract. The authors claim that the addition of dry rosemary leaves instead of fresh ones causes an increase in the antioxidant activity of active films.

Fiore et al. [39] previously reported that the coating with a polylactic acid film incorporating rosemary essential oil applied to raw chicken meat stored at 4 °C for 21 days can be a potential delivery route for providing antioxidant properties in fresh meat packaging. The combination of natural antioxidants and packaging materials may help to improve the performance of food applications while preventing oxidative reactions and increasing their action for an extended period of time [40].

#### 3.1.3. TPC in the Active Coating

TPC has been shown to be highly related to the antioxidant power of herbs [41]. The TPC of the coatings with herbs, as well as those without herbs, is presented in Table 2. The coating without any herbal additives did not contain polyphenols. It is difficult to compare the TPC observed here with that in the literature, as this value is typically shown for fresh or dry plant extracts. To the best of our knowledge, there is no information on the TPC of gelatine extracts used as an ingredient in active coatings. Moreover, TPC depends on several factors, such as harvest time, drying and extraction [42,43]. In the literature, some reports of differences in the TPC for rosemary and thyme. These values are shown to be either higher for rosemary, higher for thyme or comparable, which may also depend on the qualification of the herb (medicinal or not) [35,41]. Based on the results presented in our study, the coating containing thyme had higher polyphenol content than the one containing rosemary. For an unknown reason, it is probable that extraction from gelatine is more effective for thyme. These values are reflected in the antioxidant power of the coating. It was noted that coatings with thyme had a higher ion chelating activity than those with rosemary.

#### 3.1.4. UV-Vis Analysis of the Coatings

**Figure 4:**
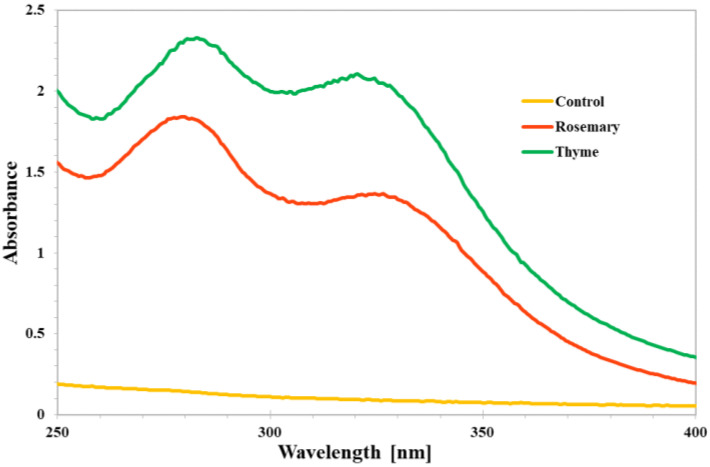
UV-Vis spectrum of coatings based on furcellaran, gelatine and herbs. Control: coating without herbs; rosemary: coatings with 5% rosemary; and thyme: coating with 5% thyme.

Transparent packaging causes the oxidation and destruction of nutritious components because light facilitates these processes. To avoid these reactions, opaque packaging and packaging containing chemicals that absorb light in the UV-Vis spectrum have been created. Plant extracts are often used to provide polymer colour and opacity, and films with plant extracts are less clear than those without. Coatings with FUR/GEL extracts containing herbs exhibited a barrier to UV (<250 nm) and visible (>300 nm) light (Figure 4).

The UV-Vis spectrum for FUR/GEL coatings with the addition of thyme shows two peaks from, among others, caffeic acid (approximately 324 nm) (Robbins, 2003) and thymol and carvacrol (approximately 282 nm) [44]). In addition, the presence of aromatic ingredients in rosemary contributes to its excellent UV barrier properties [38]. In conclusion, the addition of herbs to the coatings significantly improved their UV-barrier properties.

#### 3.1.5. Colour Parameters of the Active Coating

The results for the surface colour (L*, a* and b* values) of the coating with the incorporation of herbal extracts are shown in Table 2.

In practical applications, colour quality might influence the appearance of edible films, which, in turn, affects the acceptance of foods by consumers. The incorporation of plant extracts improves the film’s ability to block UV and visible light [45].

In several studies, it has been shown that the amount of colour change caused by plant extracts varies depending on the species and concentration of the plant extract [46]. The polyphenol content is believed to be positively correlated with colour parameters [36]. The results obtained in our study indicate that rosemary exhibited a stronger effect on a* and b* values than did thyme, despite having a slightly lower TPC.

Adding herbs extracts to the furcellaran–gelatine coatings caused a decrease in lightness change in colour towards yellow/green, which was attributed to the colour of the herbs extracts themselves. The coating with rosemary was more yellow than that with the thyme extract.

Although a general recommendation for films used in food packaging is that they should be neutral in terms of quality interference (colourless, odourless and tasteless), in some instances, the change can be perceived as positive [47]. A tendency to yellowness helped prevent the decomposition, discolouration, and off-flavours of food caused by visible light and ultraviolet light [48]. Furthermore, other researchers have claimed that the rosemary extract had a positive effect on the biopolymer coating under assessment [49].

### 3.2. Influence of Tested Films on Functional Properties and Safety of Carp Fillets

#### 3.2.1. Colour Parameters of Carp Fillet in Coatings during Storage

The appearance of the food influences the colour of the packaging, which is an essential parameter in the edible packaging production process. Plant extracts can alter the colour of coatings. The results for surface colour (L*, a* and b* values) of the carp fillets covered with the coating during storage at 4 °C for 12 d are shown in Table 2.

In general, with the application of the innovative coating, the brightness (L* value) decreased, whereas the redness (a* value) and yellowness (b* values) of the carp fillets increased compared to the control samples (without coatings). However, the carp fillet coating with the thyme extract exhibited the most significant change in terms of all the colour parameters. Similarly, Cheng et al. [50] reported that the addition of phenolic compounds darkened the edible films. In contrast, Jouki et al. [51] found an increase in the L* value during chilled storage (4 °C) of rainbow trout fillets coated with quince seed mucilage films containing thyme and oregano oils. According to Jung et al. [52], colour changes in meat and fish during storage can be caused by both enzymatic and non-enzymatic processes, resulting in myofibrillar protein breakdown and myofibril disorganisation. The colour of the film could have changed due to the oxidation of the phenolic compound in rosemary [46]. Another reason may be the type and concentration of an extract used in a certain treatment; therefore, a direct value comparison is difficult. Furthermore, according to Zeng et al. [53], the Maillard reaction between the fish protein and carbohydrates from the coating may have occurred, whereas in acidic conditions, yellow pigments may have been created, leading the degree of colour change being affected by processing conditions and moisture content.

As a result, the incorporation of plant extracts in films offers an appropriate light barrier, which is also necessary for preventing oxidation processes [54]. According to Gómez-Estaca et al. [55], films from fish skin and bovine gelatine incorporated with polyphenol-rich aqueous extracts from rosemary and oregano improve light-barrier characteristics and antioxidant activity, regardless of the type of gelatine used. Therefore, it was assumed that the use of innovative coatings based on gelatine from carp skin and herbal extracts of dark colour (thyme and rosemary) can positively affect the oxidative stability of fillets.

#### 3.2.2. Texture Profile Analyses of Carp Fillets in Coatings during Storage

According to Jouki, Mortazavi, Yazdi, Koocheki and Khazaei [51], texture measurement is a useful technique for determining the impact of preservation procedures on meat quality. Textural parameters are commonly used to assess fish quality along the fish value chain, which is most evident in the evaluation of effects caused by handling and processing methods on the shelf-life of fish products and consumer satisfaction. Freshness is closely connected to consumer perceptions of taste, texture and flavour, and it is one of the most important factors in evaluating fish quality [56]. The results of the texture profile measurement test for the analysed variants over 12 days of storage are summarised in Table 3.

There were no significant observed changes in chewiness, springiness or cohesiveness among the treated groups as the storage period was prolonged. Moreover, there was no significant difference (*p* < 0.5) in the fish hardness during storage of the control sample, or in the coatings with gelatine or *rosemary* extract. At the end of the storage period, the carp treated with the thyme extract coating demonstrated a significant reduction (*p* < 0.05) in hardness. This result was rather surprising; however, it might not have been accidental. A similar result was recently reported for stored tilapia fillets treated with a polyphenol-rich bioactive coating [57]. The decrease in hardness could be related to the breakdown and degradation of myofibrillar proteins, which make up 70–80% of the total protein content in fish muscle [58].

The adhesiveness value was −6.99 ± 0.86 on the first day following production, and the highest amount was obtained in fillet coated with the thyme extract (−4.41 ± 0.53) on day 12 of the storage period. This finding is consistent those obtained by Feng, Ng, Mikš-Krajnik and Yang [58], who investigated the effects and methods of tea polyphenol–gelatine coating spoilage and myofibril degradation in fish fillets during cold storage. Saito et al. [59] discovered that plant polyphenols have the ability to inhibit the actions of fish-derived metalloproteinase, which are responsible for the degradation of collagen and softening of flesh.

#### 3.2.3. Water Activity of Carp Fillets in Coatings during Storage

Water activity (a_w_) is a key measure for regulating the growth of microbes in stored meat and can be described as the amount of free water contained in a product that is available for bacterial growth [60]. In Table 4, the water activity noted for innovative coatings of herbal extracts during 12 day of storage is shown.

The changes in water activity of the carp fillets treated with the coatings during storage at 4 °C for 12 days are shown in Table 4. The water activity of the carp fillets treated with the FUR/GEL coatings was significantly (*p* < 0.05) increased as a result of the addition of thyme and rosemary extracts on the ninth day of storage, and the fish fillet treated with the coating with thyme extract had the highest water activity (0.967) in the group. However, no statistically significant differences in water activity were observed between the remaining storage days. These results are similar to those achieved in the research conducted by Tosati, Messias, Carvalho, Rodrigues Pollonio, Meireles and Monteiro [1], who studied the antimicrobial effects of fresh frankfurter sausages coated with turmeric starch residue and a gelatine blend. The authors did not find any significant differences in water activity among treatments or storage time. However, in their study, one of the edible coatings from polymeric matrices showed high hygroscopicity, probably because of water migration from food to film, thereby reducing the a_w_ values in the sausages. Water activity has been viewed as an important criterion for the evaluation and control of food safety and the quality of foodstuffs since the 1950s when it became obvious that water content is strongly related to microbial growth limitations. It can be summarised that the steady a_w_ values observed in our coated study groups represent a positive effect and may be related to the water vapor barrier property of gelatine material [60].

#### 3.2.4. Oxidative Stability of Carp Fillets in Active Coatings during Storage

Lipid oxidation is the main factor limiting the shelf-life of marine products. The second factor is microbiological quality [61]. The present experiment was designed to establish whether the use of the newly developed films would inhibit the oxidation process rather than microbiological growth. It was carried out to establish whether the applied coating would inhibit oxidation, which is one of the factors causing flavour deterioration. In contrary to popular opinion, some polyphenols may exhibit pro-oxidant activity [62]. Although the antioxidant activity of rosemary and thyme was proved in many other studies, we wanted to confirm that the oxidation would be inhibited in FUR/GEL coatings prepared according to our method. Quite often, in a complicated matrix, some interactions between various ingredients may result in deteriorated quality [62]. TBARSs indicate the freshness of fish products during lipid oxidation at the time of storage. The TBARS values obtained for the samples are listed in Table 4.

The TBARS values obtained for the control fillets were significantly higher (*p* < 0.05) than those for the other treatments on day 3. One of the reasons that the TBARS value increased during the storage of the control groups could be the partial dehydration of fish and enhanced oxidation of unsaturated fatty acids [63]. Surprisingly, the TBARS values for the carp fish coated without herbs were lower than those observed for the control. This shows that such a coating may be effective in protecting the fish flesh from oxidation and could be used in the case of herbal flavour not being accepted by consumers. A significant increase (*p* < 0.05) in TBARS values from 0.40 to 0.71 mg TBARS/kg on day 3 was noted for this coating. However, the effect of FUR/GEL coatings without herbs was not as notable as that of coatings with herbs, which may be related to the low antioxidant capacity of these coatings. Simultaneously, no statistically significant changes in the TBARS index were observed for the fish fillets stored with the tested coatings. Moreover, by the 12th day of storage, the control fillets and those covered in the FUR/GEL coating without herbs demonstrated a significant increase (*p* < 0.05) in their TBARS values to 1.70 ± 0.26 and 1.18^b^ ± 0.20, respectively. However, during the final days of storage, there were no significant differences in the TBARS values of the carp fillet covered with the tested coatings containing the addition of rosemary and thyme extracts. In the study conducted by Makri [64], rosemary reduced the development of thiobarbituric-acid-reactive compounds and prolonged shelf-life when minced gilthead sea bream was frozen for 3 months. The rosemary-treated samples had a lower TBARS content than the control group after 24 days of storage, indicating that rosemary demonstrates high antioxidant capacity. Choulitoudi et al. [65] reported that coatings with higher total phenol contents raise the concentration of these compounds in the fish’s muscle, providing more antioxidant protection. There was a large amount of rosmarinic acid present in the rosemary extract, which is likely responsible for the high antioxidant activity. Moreover, the preservative rosemary extract increased the acceptance that dismisses the free radical chain and, thus, retards the oxidation process because of its free radical scavenging activity, as reported by Nawaz et al. [66]. Similar results were reported by Khalafalla et al. [67] who studied the effect of thyme (0.5%) and rosemary (1.5%) dip treatments on the quality and shelf-life of Nile tilapia fillets. Both extracts had strong antioxidant activity and extend the shelf-life of fish for 9 days. Furthermore, Tural and Turhan [68] found that anchovy fillets coated with an anchovy by-product protein and thyme essential oil can inhibit lipid oxidation as a function of storage period. Oguzhan Yildiz [69] reported the effectiveness of a chitosan coating enriched with thyme oil on rainbow trout fillets and found lower TBARS contents in chitosan-coated samples with 2% thyme essential oil samples than those of the control groups throughout the storage period. The incorporation of black carrot powder (BCP) into the carboxymethyl chitosan films decreased TBARS and could also effectively delay the formation of secondary oxidation products in fried shrimp during refrigerated storage at 4 ± 1 °C for 24 days [70]. In this study, we confirmed that innovative active coatings containing a mixture of furcellaran, carp skin gelatine and herbs are effective in inhibiting lipid oxidation in fish fillets.

The use of edible films can affect the microbiological growth of food products. On the one hand, fish gelatine and herb extracts may exhibit bacteriostatic and bactericidal properties. On the other, some ingredients of edible coatings, mainly poly- and oligosaccharides, show prebiotic activity, enhancing the growth of certain bacterial strains [61]. Thus, further research should be focused on the microbial quality of food products coated with these active coatings.

### 3.3. Sensory Analysis of Carp Fish Fillets Covered with Coatings

**Figure 5:**
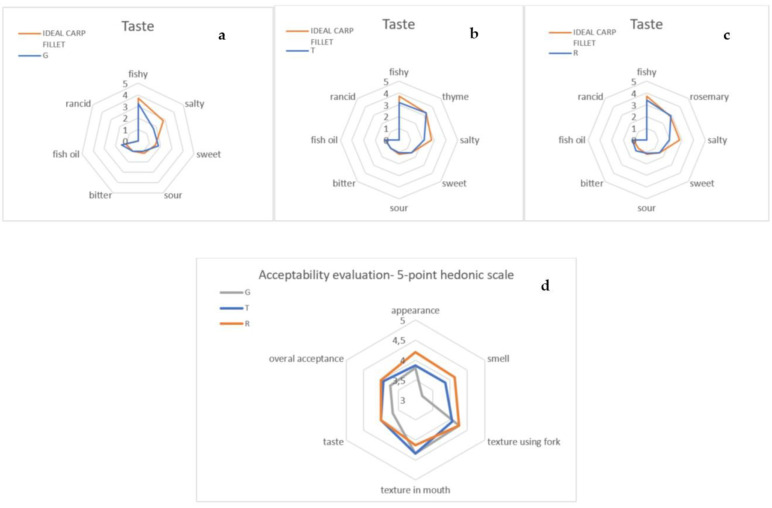
The effects of treatment on sensory evaluation of carp taste (**a**–**c**) and acceptability on 5-point hedonic scale (**d**).

The effects of treatment on the sensory evaluation of carp taste are presented in Figure 5a–c.

According to the panellists, only fish oil and rancid taste notes were the same for G, T and R, as for the ideal sample. The fish fillet covered with herbal coatings (T and R) seemed to be more salty than the fish containing gel without herbs; however, fish saltiness, in the panellists’ opinion, was lower than perfect. It is worth noting that the fish were not salted before baking; therefore, the herbs, to a small extent, emphasised the natural saltiness of the fish. Thyme and rosemary tastes were assessed as almost ideal. The fish in the gel without herbs (G) were slightly sweeter and less bitter than those in the samples with herbal coatings.

The average acceptability values for individual attributes obtained from the hedonic scale are shown in Figure 5. The results of the study reveal that the carp fish treated with rosemary extract were the most acceptable according to the panellists, especially in terms of appearance, smell and texture when using a fork. Taste acceptance evaluation was similar for the R and T samples (4); however, overall acceptability was slightly higher in the case of R fish (average, 4.25, compared to T, 4.00). Sensory and organoleptic evaluation conducted by Opeña et al. [71] also indicated that fish treated with guava leaf and lemongrass extracts were more acceptable in professional panellists’ opinions than those without plant extracts (4.45, 4.28 and 4.08, respectively). Nonetheless, the general acceptability of fish covered with calamansi leaf extract was notably lower (3.73), which indicates that evaluation scores depend on the type of extract applied. Carp fillets covered in gel without herbs (G) were rated worse among the studied samples, especially in terms of appearance, smell and taste. The overall acceptability of the G product was 3.75. Moreover, Buchtova et al. [72] stated that escolar (*Epidocybium flavobrunneum*) fish prepared in different marinates were rated notably higher than the control sample (fish marinated in herbs received the second highest score among the different marinates). These results indicate that herbal extracts can be successfully used to increase the value and acceptability of fish products covered in FUR/GEL coatings.

## 4. Conclusions

The highest antioxidant activities of rosemary and thyme extracts were achieved via water extraction; thus, to simplify the process of producing the coatings, the herbs were extracted in a carp skin gelatine solution.

The colour, texture and water activity of the carp fish treated with the FUR/GEL coating were significantly affected by the addition of the herb extracts. It was demonstrated that the carp fillets coated with rosemary and thyme extracts became slightly darker and showed a tendency towards redness and yellowness. Furthermore, based on these results, it could be concluded that the developed FUR/GEL coating with the addition of the herb extracts slowed down the processes of lipid oxidation in the carp fillets during the storage period. This also revealed that the coating could protect the fish from oxidative rancidity during refrigerated storage. In general, all treatments were well-accepted for sensory characteristics, and the fillets with 5% rosemary in the coating were regarded as better based on appearance, smell, texture (using a fork) and overall acceptability attributes evaluated by panellists. Therefore, the innovative coatings produced from carp processing waste could have high potential as components in convenience food products and could extend the shelf-life of carp fillets during refrigerated storage. However, the usage of the obtained coatings as preservatives may be limited due to their high water content, meaning that they are applicable only to water-rich products. Future research efforts should be directed towards evaluating the active effects of the obtained coatings using different types of food products. Moreover, the microbial quality of these products should also be assessed prior to commercialisation.

## Figures and Tables

**Table 1:** The antioxidant properties and colour parameters of coatings (mean values ± standard errors).

	Antioxidant Properties of Coatings		Colour Parameters		
Type of Coating	FRAP Value [mM Fe_2_SO_4_/L]	TPC [mg gallic acid/L]	L* (D65)	a* (D65)	b* (D65)
Coating without herbs	0.03 ^c^ ± 0.00	0.00 ^c^ ± 0.00	27.46 ^a^ ± 0.02	−0.26 ^c^ ± 0.01	−0.69 ^c^ ± 0.01
Coating with rosemary	0.93 ^b^ ± 0.00	276.21 ^b^ ± 3.31	24.88 ^b^ ± 0.02	0.34 ^a^ ± 0.01	1.20 ^a^ ± 0.02
Coating with thyme	0.95 ^a^ ± 0.00	290.41 ^a^ ± 5.81	23.66 ^c^ ± 0.01	0.11 ^b^ ± 0.01	0.11 ^b^ ± 0.01

Results are expressed as mean ± standard deviation a, b, and c: mean values with different superscript letters in the same columns show significant differences (*p* < 0.05)

**Table 2:** Colour value of fish fillets during storage in coatings (mean values ± standard errors).

Group	Day	L* (D65)		a* (D65)		b* (D65)	
K	0	50.76 ^bcd^	±0.25	−0.95 ^efg^	±0.17	4.53 ^f^	±0.34
	6	48.00 ^de^	±0.59	−0.06 ^cde^	±0.17	4.23 ^f^	±0.18
	9	52.60 ^ab^	±0.80	−1.29 ^fg^	±0.10	4.44 ^f^	±0.30
	12	54.52 ^a^	±0.81	−1.60 ^g^	±0.17	6.27 ^de^	±0.45
G	6	50.61 ^bcd^	±0.81	1.19 ^bc^	±0.46	7.48 ^cde^	±0.61
	9	48.15 ^de^	±0.78	−0.26	±0.36	6.03 ^e^	±0.23
	12	51.95 ^abc^	±1.56	−0.68 ^ef^	±0.26	6.99 ^cde^	±0.43
T	6	40.74 ^h^	±0.77	2.63 ^a^	±0.22	10.43 ^ab^	±0.50
	9	42.96 ^gh^	±0.61	2.06 ^ab^	±0.26	13.65 ^a^	±0.61
	12	40.53 ^h^	±0.67	3.22 ^a^	±0.28	13.53 ^a^	±0.58
R	6	44.47 ^fg^	±0.82	0.62 ^bcd^	±0.19	8.96 ^bc^	±0.33
	9	46.45 ^ef^	±0.75	−0.01 ^cde^	±0.26	7.44 ^cde^	±0.56
	12	48.86 ^cdb^	±0.36	−0.06 ^cde^	±0.14	8.23 ^bcd^	±0.23
Effect of group		***		***		***	
Effect of storage time		***		***		***	
Effect of group × time		***		***		***	

K: uncovered sample; G: sample covered in FUR/GEL coating without herbs; T: sample covered in FUR/GEL coating with 5% thyme; R: sample covered in FUR/GEL coatings with 5% rosemary. ^a–h^ Different letters in the column indicate significant differences between means (*p* < 0.05). *** Significant at *p* < 0.01.

**Table 3:** Texture profile analyses of carp fillets covered in coatings during storage (mean values ± standard errors).

Day of Storage	Group	Hardness [N]	Adhesiveness	Springiness	Cohesiveness	Chewiness
0	K	16.73 ^a^ ± 4.29	−6.99 ^abc^ ± 0.86	0.64 ^a^ ± 0.05	0.54 ^a^ ± 0.09	5.07 ^a^ ± 1.26
3		16.27 ^a^ ± 3.18	−5.84 ^ab^ ± 1.42	0.55 ^a^ ± 0.04	0.43 ^a^ ± 0.04	4.55 ^ab^ ± 0.92
6		21.65 ^a^ ± 3.01	−6.20 ^ab^ ± 1.20	0.60 ^a^ ± 0.03	0.44 ^a^ ± 0.04	6.23 ^a^ ± 0.70
9		20.77 ^a^ ± 0.90	−6.20 ^ab^ ± 0.38	0.57 ^a^ ± 0.03	0.45 ^a^ ± 0.02	5.53 ^a^ ±1.24
12		14.81 ^ab^± 1.10	−5.27 ^ab^ ± 1,21	0.58 ^a^ ± 0.02	0.46 ^a^ ± 0.03	4.03 ^ab^ ± 0.70
3	G	16.73 ^ab^ ±4.29	−7.67 ^abc^ ± 0.96	0.59 ^a^ ± 0.03	0.38 ^a^ ± 0.01	3.42 ^ab^ ± 0.11
6		17.20 ^a^± 2.67	−7.34 ^abc^ ± 1.24	0.52 ^a^ ± 0.03	0.41 ^a^ ± 0.02	3.97 ^ab^ ± 0.71
9		11.06 ^ab^ ± 0.44	−6.06 ^ab^ ± 0.62	0.56 ^a^ ± 0.02	0.44 ^a^ ± 0.01	2.76 ^ab^ ± 0.35
12		17.86 ^a^ ± 0.22 a	−7.70 ^abc^ ± 0.90	0.58 ^a^ ± 0.03	0.41 ^a^ ± 0.02	4.20 ^ab^ ± 0.24
3	T	15.75 ^ab^ ± 0.55	−8.37 ^abc^ ± 0.84	0.53 ^a^ ± 0.03	0.42 ^a^ ± 0.04	3.99 ^ab^ ± 0.67
6		12.94 ^ab^ ± 0.55	−8.54 ^abc^ ± 1.42	0.53 ^a^ ± 0.03	0.41 ^a^ ± 0.03	2.79 ^ab^ ± 1.52
9		14.63 ^a^ ± 0.43	−11.29 ^b^ ± 1.74	0.6 ^a^ ± 0.04	0.43 ^a^ ± 0.01	3.82 ^ab^ ± 0.33
12		4.56 ^b^ ± 0.14	−4.41 ^a^ ± 0.53	0.6 ^a^ ± 0.02	0.38 ^a^ ± 0.01	1.05 ^b^ ± 0.47
3	R	13.79 ^ab^ ± 1.03	−7.67 ^abc^ ± 0.96	0.59 ^a^ ± 0.03	0.38 ^a^ ± 0.02	3.98 ^ab^ ± 0.41
6		15.27 ^a^ ± 1.37	−11.84 ^c^ ± 1.11	0.59 ^a^ ± 0.01	0.44 ^a^ ± 0.02	4.80 ^ab^ ± 0.46
9		15.77 ^a^ ± 0.48	−10.44 ^bdc^ ± 0.80	0.62 ^a^ ± 0.03	0.49 ^a^ ± 0.01	3.78 ^ab^ ± 0.60
12		13.18 ^ab^ ± 0.68	−9.72 ^bdc^ ± 1.20	0.63 ^a^ ± 0.04	0.46 ^a^ ± 0.03	3.78 ^ab^ ± 0.60
Effect of group		*	***	Ns	Ns	***
Effect of storage time		Ns		Ns	Ns	Ns
Effect of group × time		Ns	***	Ns	Ns	Ns

a–d: mean values with different superscript letters in the same columns show significant differences (*p* < 0.05). K: uncovered sample; G: sample covered in FUR/GEL coating without herbs; T: sample covered with FUR/GEL coatings with 5% thyme; R: sample covered with FUR/GEL coatings with 5% rosemary. *** Significant at *p* < 0.01. * Significant at *p* < 0.05. Ns—not significant.

**Table 4:** Water activity and oxidation rate of fish fillet storage in innovative coatings (mean values ± standard errors).

Group	Day of Storage	Water Activity	TBARS [mg/kg]
K	0	0.953 ^e^ ± 0.00	0.40 ^e^ ± 0.13
	3	0.964 ^ab^ ± 0.00	1.31 ^b^ ± 0.20
	6	0.962 ^abc^ ± 0.00	1.30 ^b^ ± 0.12
	9	0.958 ^bcde^ ± 0.00	1.35 ^b^ ± 0.46
	12	0.959 ^bcde^ ± 0.00	1.70 ^a^ ± 0.26
G	3	0.954 ^de^ ± 0.00	0.71 ^cd^ ± 0.17
	6	0.959 ^abcde^ ± 0.00	0.86 ^c^ ± 0.38
	9	0.956 ^cde^ ± 0.00	0.69 ^cd^ ± 0.12
	12	0.959 ^bcde^ ± 0.00	1.18 ^b^ ± 0.20
T	3	0.958 ^bcde^ ± 0.00	0.19 ^e^ ± 0.02
	6	0.961 ^abcd^ ± 0.00	0.40 ^e^ ± 0.11
	9	0.967 ^a^ ± 0.00	0.24 ^e^ ± 0.09
	12	0.957 ^bcde^ ± 0.0	0.36 ^e^ ± 0.04
R	3	0.961 ^dbg^ ± 0.00	0.23 ^e^ ± 0.07
	6	0.957 ^bcde^ ± 0.00	0.40 ^e^ ± 0.09
	9	0.963 ^ebi^ ± 0.00	0.20 ^e^ ± 0.04
	12	0.958 ^bcde^ ± 0.00	0.44 ^de^ ± 0.05
Effect of group		***	***
Effect of storage time		***	***
Effect of group × time		***	***

Different letters in the columns indicate significant differences between means within the group (*p* < 0.05). *** Significant at *p* < 0.01.

## Data Availability

The datasets generated during the current study are available from the corresponding author upon reasonable request.
